# Sex‐Specific Cross‐Sectional Associations and Exploratory Mediation Analysis of Cardiovascular Disease Risk by Neutrophil‐to‐Lymphocyte Ratio in Cadmium‐Exposed Populations

**DOI:** 10.1155/crp/3957573

**Published:** 2026-10-21

**Authors:** Xinyi Li, Kaiwen Wang, Xu Hui, Ruiqi Yang, Weize Kong, Liangying Hou, Siyu Zhang, Peijing Yan, Yongbin Lu, Xiaowei Niu

**Affiliations:** ^1^ The First School of Clinical Medicine Lanzhou University Lanzhou 730000 Gansu China lzu.edu.cn; ^2^ Evidence-Based Social Science Research Center Health Technology Assessment Center School of Public Health Lanzhou University Lanzhou Gansu China lzu.edu.cn; ^3^ Gansu Key Laboratory of Evidence-Based Medicine Lanzhou University Lanzhou Gansu Province 730000 China lzu.edu.cn; ^4^ Chinese Center for Disease Control and Prevention National Institute for Occupational Health and Poison Control Beijing China chinacdc.cn; ^5^ Clinical Research Center Sichuan Provincial People’s Hospital University of Electronic Science and Technology of China Chengdu Sichuan China uestc.edu.cn; ^6^ School of Public Health Lanzhou University Lanzhou Gansu China lzu.edu.cn; ^7^ Department of Cardiology The First Hospital of Lanzhou University Lanzhou Gansu China lzu.edu.cn; ^8^ Gansu Key Laboratory of Cardiovascular Diseases The First Hospital of Lanzhou University Lanzhou Gansu China lzu.edu.cn; ^9^ Gansu Clinical Medical Research Center for Cardiovascular Diseases The First Hospital of Lanzhou University Lanzhou Gansu 730000 China lzu.edu.cn

**Keywords:** cadmium, cardiovascular disease, dose–response relationship, mediation effects, neutrophil-to-lymphocyte ratio

## Abstract

**Background:**

Cardiovascular disease (CVD) remains the leading cause of mortality worldwide, with its burden continuing to rise. Environmental cadmium (Cd) exposure has emerged as a potential risk factor for CVD. This study aimed to investigate the associations between Cd exposure and CVD risk, and to explore cross‐sectional associations and a potential inflammatory pathway using exploratory mediation analysis.

**Methods:**

This study utilized data from the National Health and Nutrition Examination Survey. Logistic regression models were used to identify factors influencing CVD risk. Dose–response relationships were evaluated using restricted cubic splines (RCS). Exploratory mediation analysis was conducted to assess the potential mediating role of NLR in the association between Cd exposure and CVD risk.

**Results:**

Individuals with CVD had significantly higher levels of blood Cd, urinary Cd, and NLR than controls (*p* < 0.05) in both sexes. In males, the highest quartile (Q4) of blood Cd (OR = 3.06, 95% CI: 1.45‐6.46, *p* = 0.004) and urinary Cd (OR = 3.02, 95% CI: 1.52–6.02, *p* = 0.002) were significantly associated with higher CVD prevalence. Conversely, no significant association was observed in females for blood Cd (*p* = 0.505), and the association for urinary Cd (*p* = 0.046) was notably weaker and less robust than that in males. Interaction analyses confirmed sex‐specific differences (p‐interaction < 0.05). NLR showed significant exploratory mediating effects (indirect effects), accounting for 9.4% (95% CI: 3.5%–15.2%) of the blood Cd–CVD link and 5.0% (95% CI: 1.9%–8.1%) of the urinary Cd–CVD link in males. Similar significant mediation was observed in the general population for blood Cd: 7.2% (95% CI: 2.2%–12.2%), and for urinary Cd: 6.4% (95% CI: 2.8%–10.0%).

**Conclusions:**

This cross‐sectional study identified significant sex‐specific and dose‐dependent associations between Cd exposure and CVD risk. Inflammatory responses, particularly those reflected by elevated NLR, were cross‐sectionally associated with the observed Cd–CVD link, suggesting a potential pathway that warrants prospective validation, especially in males.

## 1. Introduction

Environmental pollution, particularly heavy metal contamination, has emerged as a significant global public health challenge. With the rapid advancement of industrialization, the concentrations of heavy metals such as cadmium (Cd), lead, arsenic, and mercury in the air, water, and soil have been steadily increasing. These toxic metals pose persistent threats to human health due to their long‐term environmental persistence and bioaccumulation potential [1]. Research indicates that heavy metals can enter the human body through various pathways, including inhalation, ingestion, and dermal absorption, and accumulate in the body, contributing to the onset and progression of chronic diseases, notably cardiovascular diseases (CVD) [2, 3]. Consequently, exploring the relationships between heavy metal exposure and human health, along with the underlying mechanisms, is crucial for informing effective environmental protection policies and advancing public health strategies.

CVD is one of the leading causes of death worldwide. According to the World Health Organization, CVD accounts for approximately 32% of global deaths [4]. In the United States, statistical data from the American Heart Association in 2018 indicated that 127 million adults, nearly half of the population, were affected by CVD [5]. Increasing evidence suggests that environmental heavy metal pollution is closely associated with the occurrence of CVD. Among these pollutants, Cd is a common environmental contaminant found extensively in natural and industrial settings. Cd is characterized by its high bioaccumulation potential and long‐term retention in the human body, posing significant risks to the cardiovascular system as demonstrated in numerous studies [2, 6]. A recent meta‐analysis confirmed that both blood Cd and urinary Cd levels were significantly associated with an increased risk of CVD, with a linear dose–response relationship observed between blood Cd levels and CVD risk [6]. Moreover, sex differences may influence the toxic effects of Cd, although previous findings on its cardiovascular impacts across sexes remain inconsistent. However, the meta‐analysis did not further investigate the sex‐specific effects of Cd on CVD or the potential underlying mechanisms.

Long‐term Cd exposure can activate the immune system, promote the release of inflammatory factors, and induce chronic low‐grade inflammation [7]. Inflammation is widely recognized as a critical factor in the occurrence and progression of CVD [8]. Elevated inflammatory markers often reflect pathological changes in the cardiovascular system. For instance, studies have reported that increased levels of inflammatory markers such as C‐reactive protein (CRP) and interleukin‐6 are strongly associated with a heightened risk of CVD [9, 10]. Among these markers, the neutrophil‐to‐lymphocyte ratio (NLR) has gained attention as a simple and easily accessible indicator of systemic inflammation. Recent research has demonstrated that NLR is closely linked to the occurrence, progression, and prognosis of CVD [11, 12]. High NLR levels typically signify a sustained inflammatory response, creating a favorable environment for the development of CVD.

Mechanistically, Cd may contribute to the development of CVD by promoting chronic inflammation [13]. However, studies examining the relationships between Cd, NLR, and CVD remain limited, particularly regarding sex‐specific differences. In this study, data from the National Health and Nutrition Examination Survey (NHANES) were utilized to identify factors influencing CVD and to analyze the effects of Cd exposure on CVD risk across sexes, as well as the associated dose–response relationships. Additionally, an exploratory mediation model was employed to assess whether the cross‐sectional association between Cd exposure and CVD risk might be partially explained by inflammatory markers, acknowledging the limitations of temporal inference in a cross‐sectional design. The aim of this study is to elucidate the intricate relationships between environmental Cd exposure, inflammatory markers, and CVD, thereby providing a scientific foundation for the prevention and management of CVD in the future.

## 2. Methods

### 2.1. Inclusion Criteria for Subjects in the NHANES Database

This analysis utilized NHANES data collected from 1999 to 2020 to investigate the association between blood and urinary Cd levels and CVD. Participants were selected based on the following inclusion and exclusion criteria. Inclusion criteria were defined as follows: participants aged 18 years and older with complete data for the variables of interest. Exclusion criteria were defined as follows: (1) age under 18 years; (2) missing data on CVD status; (3) missing data on blood Cd or urinary Cd levels; (4) missing demographic information, such as sex, race, or education level; (5) missing data on metabolic and health indicators, including body mass index (BMI), total cholesterol (TC), triglycerides (TG), low‐density lipoprotein cholesterol (LDL‐C), high‐density lipoprotein cholesterol (HDL‐C), and uric acid (UA); (6) missing data on inflammatory indices, such as CRP, NLR, systemic immune‐inflammation index (SII), platelet‐to‐lymphocyte ratio (PLR), and neutrophil‐to‐albumin ratio (NPAR). After applying these criteria, a total of 6122 participants were included in the final analysis. The study design and participant selection process are detailed in the Supporting Information (available here). Due to the unavailability of routine CRP data in the NHANES public‐use files for cycles 2011–2012 and 2013–2014, these two cycles were excluded from the present analysis. The final pooled dataset included cycles from 1999–2010, 2015–2016, and 2017–2020.

### 2.2. Definition of CVD in the NHANES Database

In the NHANES questionnaire, participants were asked whether they had ever been diagnosed with any of the following conditions: coronary heart disease (CHD), heart failure (HF), angina pectoris, or myocardial infarction (MI). Participants who answered “yes” to any of these conditions were classified as CVD patients, while those who answered “no” to all were categorized as normal individuals.

### 2.3. Grouping Variables in the NHANES Database

Participants were categorized into groups based on thresholds derived from prior studies and clinical guidelines. Blood Cd: Levels ≥ 2 μg/L were classified as the high blood Cd group; urinary Cd: Levels ≥ 3 μg/g creatinine were used to define the high urinary Cd group [14]. For statistical normalization, blood Cd and urinary Cd values were log‐transformed, using 0.301 μg/L and 0.477 μg/g creatinine as respective boundary values. TC ≥ 6.2 mmol/L: high TC group. TG ≥ 2.3 mmol/L: high TG group. LDL‐C ≥ 4.1 mmol/L: high LDL group. HDL‐C ≥ 1.0 mmol/L: high HDL group. UA ≥ 7.0 mg/dL in males or UA ≥ 6.0 mg/dL in females: high UA group. CRP ≥ 10.0 mg/L: high CRP group. NLR ≥ 2.0: high NLR group [15]. SII ≥ 500: high SII group [16]. PLR ≥ 100: high PLR group [17]. NPAR ≥ 3.0: high NPAR group [18]. For each marker, participants with values below the threshold were classified as normal. Hypertension: Defined by self‐reported history of hypertension, chronic use of antihypertensive medications, systolic blood pressure (SBP) ≥ 140 mmHg, or diastolic blood pressure (DBP) ≥ 90 mmHg. Diabetes mellitus (DM): Defined by self‐reported diagnosis, use of prescription medication for diabetes, glycated hemoglobin (HbA1c) ≥ 6.5%, or fasting blood glucose (FBG)≥ 126 mg/dL.

### 2.4. Statistical Analysis

Participants with missing data on the primary exposure (blood or urinary Cd), primary outcome (CVD status), or key demographic variables were excluded (as detailed in Section 2.1). For the remaining analytic sample, missing values in prespecified covariates (e.g., smoking status, alcohol consumption) were rare (< 5%) and were imputed using the mean of nonmissing values to preserve sample size.

Categorical data are presented as numbers (percentages), and continuous data are expressed as mean ± standard deviation or median (interquartile range), as appropriate. Group comparisons were performed using the Rao‐Scott chi‐square test for categorical variables and survey‐weighted linear regression for continuous variables, accounting for the complex survey design. Specifically, the complex survey design was specified using the svydesign function from the R survey package, incorporating the primary sampling unit (variable: SDMVPSU), stratification (variable: SDMVSTRA), and customized sampling weights (variable: nhs_wt) constructed for multicycle analysis.

Due to a substantial amount of missing data in urinary Cd and other biomarkers, the full‐sample Mobile Examination Center (MEC) weights were applied across all participants instead of the subsample‐specific weights (e.g., WTSA2YR for urinary metals). Multicycle weights were constructed following the CDC NHANES weighting tutorial [19]. Specifically, for participants from 1999–2002 (2 cycles), the 4‐year MEC weight (WTMEC4YR) multiplied by 2/8.6 was used; for 2017–2020 (a continuous 3.2‐year prepandemic sample), the continuous MEC weight (WTMECPRP) multiplied by 1.6/8.6 was used; and for other standard 2‐year cycles (2003–2004, 2005–2006, 2007–2008, 2009–2010, and 2015–2016), the 2‐year MEC weight (WTMEC2YR) multiplied by 1/8.6 was used. All subsequent analyses, including svyglm for regression, were performed on this design object. All analyses incorporated these sampling weights, stratification, and clustering variables to account for the complex, multistage survey design of NHANES and to generate nationally representative estimates. The association between Cd levels and CVD risk was examined using multivariable logistic regression, with Cd levels modeled in quartiles; a trend test was conducted to evaluate potential dose–response patterns.

The dose–response relationship was further assessed using restricted cubic spline (RCS) regression within the survey framework, with knots placed at the 5th, 50th, and 95th percentiles of the Cd distribution. Mediation analysis was performed to assess whether the association between Cd (independent variable) and CVD (dependent variable) was mediated by the NLR (mediating variable). To account for the complex survey design, both the mediator model (NLR ∼ Cd) and the outcome model (CVD ∼ NLR + Cd) were fitted using survey‐weighted generalized linear models (svyglm) incorporating the combined examination weight (nhs_wt), stratification (SDMVSTRA), and primary sampling unit (SDMVPSU). Variance estimation was conducted using Rao–Wu bootstrap resampling with 1000 replicates via the as.svrepdesign and withReplicates functions. The indirect effect was derived as the product of coefficients (*α* × *β*), and the relative effect (proportion mediated) was calculated as indirect/total. Both models were adjusted for the same set of prespecified covariates to ensure a consistent adjustment set. Given the cross‐sectional nature of NHANES, all reported associations are observational, and the mediation analysis is exploratory and does not imply causality.

All statistical tests were two‐sided, with a *p* value < 0.05 considered significant. Analyses were conducted using *R* software (Version 4.3.0; *R* Foundation for Statistical Computing).

## 3. Results

### 3.1. Clinical and Demographic Characteristics of Study Participants

Table 1 presents the demographic characteristics of the 6122 participants in this study, including 2983 males and 3139 females. Among these, 318 (10.66%) males and 201 (6.40%) females were diagnosed with CVD. Compared to the control group from the general population, CVD patients exhibited significantly higher levels of CRP, NLR, SII, blood Cd, and urinary Cd (*p* < 0.05). Similarly, both male and female CVD patients had significantly elevated levels of NLR, SII, blood Cd, and urinary Cd compared to their respective control groups (*p* < 0.05).

**TABLE 1:** Demographic and clinical characteristics according to CVD status in NHANES.

Characteristics	Total			Male			Female		
	CVD *n* = 519	Control *n* = 5603	*p* value	CVD *n* = 318	Control *n* = 2665	*p* value	CVD *n* = 201	Control *n* = 2938	*p* value
Age, years, mean (SE)	63.95 ± 0.87	45.48 ± 0.36	< 0.001	64.24 ± 1.14	44.68 ± 0.44	< 0.001	63.50 ± 1.36	46.21 ± 0.51	< 0.001

*Race, n (%)*									
Mexican American	61 (11.75)	1105 (19.72)	< 0.001	37 (11.64)	533 (20.00)	< 0.001	24 (11.94)	572 (19.47)	0.069
Non‐Hispanic white	292 (56.26)	2397 (42.78)		198 (62.26)	1136 (42.63)		94 (46.77)	1261 (42.92)	
Non‐Hispanic black	93 (17.92)	1160 (20.71)		46 (14.46)	558 (20.94)		47 (23.38)	602 (20.49)	
Other	73 (14.07)	941 (16.79)		37 (11.64)	438 (16.43)		36 (17.91)	503 (17.12)	

*Education level, n (%)*									
Less than high school degree	192 (36.99)	1469 (26.22)	< 0.001	102 (32.08)	749 (28.11)	0.209	90 (44.78)	720 (24.51)	< 0.001
High school degree	130 (25.05)	1353 (24.15)		70 (22.01)	686 (25.74)		60 (29.85)	667 (22.70)	
More than high school degree	197 (37.96)	2781 (49.63)		146 (45.90)	1230 (46.15)		51 (25.37)	1551 (52.79)	
BMI, kg/m^2^, mean (SE)	29.55 ± 0.31	28.30 ± 0.17	< 0.001	29.28 ± 0.39	28.14 ± 0.20	0.001	29.99 ± 0.60	28.45 ± 0.22	0.002

*Smoking status, n (%)*									
Never smoker	198 (38.15)	3073 (54.85)	< 0.001	83 (26.10)	1228 (46.08)	< 0.001	115 (57.21)	1845 (62.80)	0.017
Former smoker	229 (44.12)	1358 (24.24)		172 (54.09)	770 (28.89)		57 (28.36)	588 (20.01)	
Current smoker	92 (17.73)	1172 (20.91)		63 (19.81)	667 (25.03)		29 (14.43)	505 (17.19)	

*Drinking status, n (%)*									
Never drinker	452 (87.09)	4955 (88.43)	< 0.001	295 (92.77)	2461 (92.35)	0.477	157 (78.10)	2494 (84.89)	< 0.001
Former drinker	50 (9.63)	331 (5.91)		16 (5.03)	115 (4.32)		34 (16.92)	216 (7.35)	
Current drinker	17 (3.28)	317 (5.66)		7 (2.20)	89 (3.33)		10 (4.98)	228 (7.76)	

*Physical exercise, n (%)*									
Never	384 (73.99)	3719 (66.38)	< 0.001	237 (74.53)	1758 (65.97)	0.005	147 (73.13)	1961 (66.75)	0.026
Occasionally	86 (16.57)	1375 (24.54)		54 (16.98)	664 (24.92)		32 (15.92)	711 (24.20)	
Often	49 (9.44)	509 (9.08)		27 (8.49)	243 (9.11)		22 (10.95)	266 (9.05)	
SBP, mean (SE)	127.12 ± 1.74	117.45 ± 0.66	< 0.001	125.49 ± 2.02	120.15 ± 0.85	0.009	129.70 ± 2.84	114.98 ± 0.95	< 0.001
DBP, mean (SE)	68.22 ± 1.11	69.29 ± 0.40	0.006	69.10 ± 1.45	71.51 ± 0.52	0.001	66.82 ± 1.78	67.27 ± 0.56	0.159

*hypertension, n (%)*									
No	128 (24.66)	3477 (62.06)	< 0.001	83 (26.10)	1635 (61.35)	< 0.001	45 (22.39)	1842 (62.70)	< 0.001
Yes	391 (75.34)	2126 (37.94)		235 (73.90)	1030 (38.65)		156 (77.61)	1096 (37.30)	
HbA1c, mean (SE)	5.95 ± 0.07	5.46 ± 0.02	< 0.001	6.01 ± 0.11	5.50 ± 0.02	< 0.001	5.87 ± 0.08	5.43 ± 0.03	< 0.001
FBG, mean (SE)	118.39 ± 3.93	102.69 ± 0.52	< 0.001	123.14 ± 5.77	105.90 ± 0.73	< 0.001	110.90 ± 2.49	99.75 ± 0.72	< 0.001

*Diabetes, n (%)*									
No	310 (59.73)	4778 (85.28)	< 0.001	197 (61.95)	2230 (83.68)	< 0.001	113 (56.22)	2548 (86.73)	< 0.001
Yes	209 (40.27)	825 (14.72)		121 (38.05)	435 (16.32)		88 (43.78)	390 (13.27)	
TC, mean (SE)	4.86 ± 0.08	5.10 ± 0.02	< 0.001	4.62 ± 0.09	5.06 ± 0.03	< 0.001	5.22 ± 0.12	5.13 ± 0.03	0.408
LDL‐C, mean (SE)	2.78 ± 0.07	3.07 ± 0.02	< 0.001	2.61 ± 0.08	3.13 ± 0.13	< 0.001	3.05 ± 0.12	3.02 ± 0.03	0.156
TG, mean (SE)	1.67 ± 0.06	1.39 ± 0.02	< 0.001	1.73 ± 0.09	1.49 ± 0.03	< 0.001	1.58 ± 0.07	1.30 ± 0.02	< 0.001
HDL‐C, mean (SE)	1.31 ± 0.03	1.39 ± 0.01	< 0.001	1.21 ± 0.04	1.25 ± 0.01	0.017	1.45 ± 0.04	1.52 ± 0.01	0.003

*Hyperlipidemia*									
No	316 (60.89)	3827 (68.30)	< 0.001	186 (58.49)	1702 (63.86)	0.069	130 (64.68)	2125 (72.33)	0.024
Yes	203 (39.11)	1776 (31.70)		132 (41.51)	963 (36.14)		71 (35.32)	813 (27.67)	
UA, mean (SE)	5.97 ± 0.09	5.33 ± 0.03	< 0.001	6.27 ± 0.10	6.06 ± 0.05	< 0.001	5.49 ± 0.11	4.66 ± 0.03	< 0.001

*Family history of CHD, n (%)*									
No	399 (76.88)	4933 (88.04)	< 0.001	256 (80.50)	2370 (88.93)	< 0.001	143 (71.14)	2563 (87.24)	< 0.001
Yes	120 (23.12)	670 (11.96)		62 (19.50)	295 (11.07)		58 (28.86)	375 (12.76)	
CRP, mean (SE)	1.64 ± 0.18	1.15 ± 0.07	0.010	1.56 ± 0.27	0.98 ± 0.08	0.016	1.76 ± 0.29	1.30 ± 0.10	0.202
NLR, mean (SE)	2.75 ± 0.12	2.19 ± 0.02	< 0.001	2.65 ± 0.10	2.18 ± 0.03	< 0.001	2.91 ± 0.24	2.21 ± 0.03	< 0.001
SII, mean (SE)	651.74 ± 31.14	569.80 ± 7.23	< 0.001	599.52 ± 25.50	525.85 ± 9.56	< 0.001	734.19 ± 66.82	610.02 ± 8.96	0.023
PLR, mean (SE)	152.45 ± 6.83	143.40 ± 1.56	0.179	137.46 ± 4.53	134.70 ± 2.02	< 0.001	176.13 ± 14.31	151.37 ± 1.36	0.089
NPAR, mean (SE)	9.59 ± 1.15	12.63 ± 0.34	< 0.001	9.09 ± 1.55	11.32 ± 0.46	0.318	10.39 ± 1.53	13.83 ± 0.46	< 0.001
Blood Cd, ug/L, mean (SE)	0.69 ± 0.02	0.57 ± 0.01	< 0.001	0.70 ± 0.03	0.54 ± 0.01	< 0.001	0.66 ± 0.03	0.61 ± 0.01	< 0.001
Urinary Cd, ug/g, mean (SE)	0.65 ± 0.02	0.44 ± 0.01	< 0.001	0.63 ± 0.03	0.32 ± 0.01	< 0.001	0.67 ± 0.03	0.55 ± 0.01	< 0.001

*Note:* CVD, cardiovascular disease; HbA1c, hemoglobin A1c; FBG: fasting blood glucose; TG: triglyceride; NLR, neutrophil‐to‐lymphocyte ratio; SII, systemic immune‐inflammation index; NPAR, neutrophil‐to‐albumin ratio; Cd, Cadmium.

Abbreviations: BMI = body mass index, CRP = C‐reactive protein, DBP = diastolic blood pressure, HDL‐C = high‐density lipoprotein cholesterol, LDL‐C: low‐density lipoprotein cholesterol, PLR = platelet‐to‐lymphocyte ratio, SBP = systolic blood pressure, SE = standard error, TC = total cholesterol, UA = uric acid.

### 3.2. Sex‐Specific Associations Between Cd and CVD Risk

Univariate logistic regression analysis revealed that NLR (OR = 2.20, 95% CI: 1.62–2.98, *p* < 0.001), SII (OR = 1.54, 95% CI: 1.15–2.07, *p* = 0.004), blood Cd (OR = 1.59, 95% CI: 1.05–2.41, *p* = 0.027), and urinary Cd (OR = 2.81, 95% CI: 1.93–4.12, *p* < 0.001) were all significantly associated with higher odds of CVD in the general population. Similarly, NLR (OR = 2.37, 95% CI: 1.61–3.48, *p* < 0.001), SII (OR = 1.71, 95% CI: 1.14–2.57, *p* = 0.010), blood Cd (OR = 1.85, 95% CI: 1.10–3.09, *p* = 0.020), and urinary Cd (OR = 4.63, 95% CI: 2.95–7.25, *p* < 0.001) were significantly associated with higher odds of CVD in males. Nevertheless, only NLR (OR = 1.96, 95% CI: 1.27–3.03, *p* = 0.003), SII (OR = 1.53, 95% CI: 1.00–2.36, *p* = 0.049), and urinary Cd (OR = 1.98, 95% CI: 1.15–3.40, *p* = 0.014) were significantly associated with higher odds of CVD in females (see Supporting Information available here).

To determine whether Cd is independently associated with CVD risk, multivariate logistic regression analysis was conducted (Tables 2 and 3). Compared with the first quartile (Q1) group, elevated blood Cd levels in the fourth quartile (Q4) were associated with higher odds of CVD in both the general population (OR = 2.60, 95% CI: 1.55–4.37, *p* < 0.001) and in males (OR = 3.06, 95% CI: 1.45–6.46, *p* = 0.004), with significant upward trends (*p* = 0.001 for the general population and *p* = 0.006 for males), after adjusting for confounders including age, sex, race, education level, smoking status, alcohol consumption, hypertension, DM, TC, TG, LDL‐C, HDL‐C, and UA (Table 2). Similarly, higher urinary Cd levels in the Q4 group were significantly associated with higher odds of CVD in both the general population (OR = 3.22, 95% CI: 1.76–5.98, *p* < 0.001) and in males (OR = 3.02, 95% CI: 1.52–6.02, *p* = 0.002), with significant upward trends (p < 0.001 for the general population and *p* = 0.002 for males) (Table 3). However, no significant association was observed between blood Cd and CVD risk in females (OR = 1.39, 95% CI: 0.52–3.73, *p* = 0.505). For urinary Cd, although a borderline significant association was detected (OR = 3.57, 95% CI: 1.02–12.50, *p* = 0.046), the exceedingly wide confidence interval (CI) and lack of a significant dose–response trend (*p* = 0.200) render this finding inconclusive.

**TABLE 2:** OR (95% CI) of CVD under different quantiles of blood Cd.

Participants	Blood Cd (ug/L)	Model 1	*p* value	Model 2	*p* value	Model 3	*p* value
Total (*n* = 6122)	Q1 (< 0.32)	1 (reference)		1 (reference)		1 (reference)	
	(*n* = 1503)						
	Q2 (0.32–0.56)	1.27 (0.82–1.97)	0.286	1.05 (0.66–1.67)	0.843	1.08 (0.68–1.73)	0.743
	(*n* = 1594)						
	Q3 (0.56–0.78)	2.42 (1.57–3.73)	< 0.001	1.83 (1.14–2.94)	0.013	1.83 (1.11–3.03)	0.019
	(*n* = 1554)						
	Q4 (> 0.78)	2.74 (1.84–4.08)	< 0.001	2.31 (1.50–3.58)	< 0.001	2.60 (1.55–4.37)	< 0.001
	(*n* = 1471)						
	*p*_trend_	< 0.001		< 0.001		0.001	

Male (*n* = 2983)	Q1 (< 0.30)	1 (reference)		1 (reference)		1 (reference)	
	(*n* = 867)						
	Q2 (0.30–0.51)	1.33 (0.82–2.15)	0.239	0.94 (0.57–1.55)	0.796	0.90 (0.51–1.57)	0.707
	(*n* = 758)						
	Q3 (0.51–0.78)	3.57 (2.08–6.11)	< 0.001	2.38 (1.34–4.24)	0.003	2.14 (1.08–4.27)	0.030
	(*n* = 648)						
	Q4 (> 0.78)	3.79 (2.26–6.36)	< 0.001	3.03 (1.74–5.29)	< 0.001	3.06 (1.45–6.46)	0.004
	(*n* = 710)						
	*p*_trend_	< 0.001		< 0.001		0.006	

Female (*n* = 3139)	Q1 (< 0.38)	1 (reference)		1 (reference)		1 (reference)	
	(*n* = 636)						
	Q2 (0.38–0.59)	1.24 (0.54–2.81)	0.609	1.07 (0.45–2.52)	0.882	1.13 (0.50–2.55)	0.766
	(*n* = 836)						
	Q3 (0.59–0.78)	1.67 (0.84–3.33)	0.143	1.18 (0.56–2.46)	0.666	1.14 (0.54–2.44)	0.724
	(*n* = 906)						
	Q4 (> 0.78)	1.80 (0.86–3.80)	0.119	1.44 (0.66–3.14)	0.364	1.39 (0.52–3.73)	0.505
	(*n* = 761)						
	*p*_trend_	0.300		0.700		0.900	

*Note:* Model 1: unadjusted; Model 2: adjusted for age; Model 3: additionally adjusted for education level, race, smoking status, drinking status, hypertension, diabetes mellitus, family history of CHD, TC, TG, LDL‐C, HDL‐C, and UA.

**TABLE 3:** OR (95% CI) of CVD under different quantiles of urinary Cd.

Participants	Urinary Cd (ug/g)	Model 1	*p* value	Model 2	*p* value	Model 3	*p* value
Total (*n* = 6122)	Q1 (< 0.22)	1 (reference)		1 (reference)		1 (reference)	
	(*n* = 1426)						
	Q2 (0.22–0.47)	1.67 (0.92–3.02)	0.899	1.42 (0.79–2.55)	0.238	1.46 (0.77–2.78)	0.242
	(*n* = 1545)						
	Q3 (0.47–0.72)	3.03 (1.71–5.36)	< 0.001	2.45 (1.32–4.54)	0.005	2.52 (1.37–4.63)	0.003
	(*n* = 1568)						
	Q4 (> 0.72)	4.88 (2.98–8.00)	< 0.001	3.39 (1.89–6.08)	< 0.001	3.22 (1.76–5.98)	< 0.001
	(*n* = 1583)						
	*p*_trend_	< 0.001		< 0.001		< 0.001	

Male (*n* = 2983)	Q1 (< 0.12)	1 (reference)		1 (reference)		1 (reference)	
	(*n* = 985)						
	Q2 (0.12–0.36)	1.30 (0.74–2.26)	0.359	0.87 (0.50–1.53)	0.638	0.88 (0.46–1.67)	0.685
	(*n* = 803)						
	Q3 (0.36–0.62)	3.49 (1.79–6.81)	< 0.001	2.08 (0.98–4.46)	0.058	2.22 (1.03–4.75)	0.041
	(*n* = 640)						
	Q4 (> 0.62)	8.30 (5.10–13.50)	< 0.001	3.91 (2.15–7.11)	< 0.001	3.02 (1.52–6.02)	0.002
	(*n* = 555)						
	*p*_trend_	< 0.001		< 0.001		0.002	

Female (*n* = 3139)	Q1 (< .33)	1 (reference)		1 (reference)		1 (reference)	
	(*n* = 441)						
	Q2 (0.33‐0.57)	4.51 (1.38–14.70)	0.013	3.59 (1.11–11.60)	0.034	3.73 (1.10–12.60)	0.035
	(*n* = 742)						
	Q3 (0.57‐0.80)	5.77 (2.06–16.10)	< 0.001	3.64 (1.25–10.60)	0.018	3.10 (1.04–9.31)	0.043
	(*n* = 928)						
	Q4 (> 0.80)	6.78 (2.38–19.30)	< 0.001	3.56 (1.15–11.10)	0.028	3.57 (1.02–12.50)	0.046
	(*n* = 1028)						
	*p*_trend_	0.002		0.120		0.200	

*Note:* Model 1: unadjusted; Model 2: adjusted for age; Model 3: additionally adjusted for education level, race, smoking status, drinking status, hypertension, diabetes mellitus, family history of CHD, TC, TG, LDL‐C, HDL‐C, and UA.

To test outcome heterogeneity, we repeated the survey‐weighted quasibinomial models for MI, HF, CHD, and stable angina pectoris (SAP) individually, using the same NHANES complex‐survey framework (sampling weights nhs_wt as constructed in the Methods section, strata SDMVSTRA, PSU SDMVPSU; all estimates derived from the design object) (Supporting Information available here).

Consistent with the composite CVD signal, MI carried the strongest and most consistent dose–response association. Under the fully specified models, the highest versus lowest blood Cd quartile was associated with MI risk (OR = 6.25, 95% CI: 3.35–11.70, *p* < 0.001), and the corresponding urinary Cd (OR = 5.12, 95% CI: 2.43–10.80, *p* < 0.001), both with significant ordinal trends (*p* < 0.001 for the Cd trend). HF also showed a clear positive gradient for blood Cd (OR = 3.61, 95% CI: 1.77–7.36, *p* < 0.001), whereas the urinary‐Cd association for HF was meaningfully attenuated (adjusted *p* = 0.184) and should be interpreted cautiously.

CHD and SAP showed weaker, largely nonsignificant signals after full adjustment, suggesting that the composite CVD signal reflects severe/definitive diagnoses. As expected for rare self‐reported outcomes in a complex survey, some strata were sparse. Notably, in the urinary‐Cd model among women, the CHD referent cell (Q1) contained zero events, precluding a finite OR (95% CI). We, therefore, used Q2 as the reference, though this substitution may reduce estimate stability. The sparsity is flagged in the footnote, with events/total reported per quartile.

To formally evaluate whether sex modifies the association between Cd and CVD risk, we tested the interaction terms between Cd levels and sex in survey‐weighted logistic regression models. As shown in Table 4, the interactions were statistically significant for both blood Cd (*p* = 0.012) and urinary Cd (*p* < 0.001). These results indicate that the effect of Cd differs significantly by sex. The association was markedly stronger in males, whereas in females, the wide CIs likely reflect limited statistical power rather than a true null association.

**TABLE 4:** Interaction analysis between cadmium exposure and sex on cardiovascular disease risk.

Exposure	Variable	OR (95% CI)	*p* value
Blood Cd (log10)	Blood Cd	5.24 (2.70–10.18)	< 0.001
	Sex (female vs. male)	1.27 (0.62–2.59)	0.518
	Blood Cd × Female (*n* = 1028)	0.32 (0.13–0.77)	0.012

Urine Cd (log10)	Urine Cd	6.19 (3.52–10.87)	< 0.001
	Sex (female vs. male)	1.34 (0.72–2.48)	0.351
	Urine Cd × Female	0.23 (0.11–0.48)	< 0.001

*Note:* Adjusted for age, race, education level, smoking status, drinking status, hypertension, diabetes mellitus, family history of CHD, TC, TG, LDL‐C, HDL‐C, and UA.

Abbreviations: CI = confidence interval, OR = odds ratio.

### 3.3. Dose–Response Relationship Between Cd and CVD Risk by Sex

RCS analysis was performed to examine the linear and nonlinear dose–response relationships between Cd levels and CVD risk (Figure 1). A significant positive linear association was observed between blood Cd levels and CVD risk in both the general population (*p*_overall_ < 0.001, *p*_nonlinear_ = 0.163), and in males (*p*_overall_ < 0.001, *p*_nonlinear_ = 0.106), with the risk showing a near‐linear increase across the exposure range. For urinary Cd, the association with CVD risk was also positive and linear in the general population (*p*_overall_ < 0.001, *p*_nonlinear_ = 0.559). However, in males, the relationship exhibited a distinct J‐shaped pattern, with risk remaining relatively stable at lower urinary Cd concentrations and rising sharply beyond an inflection point. In females, no significant dose–response relationship was found for either blood Cd (*p*_overall_ = 0.123) or urinary Cd (*p*_overall_ = 0.180).

**FIGURE 1:**
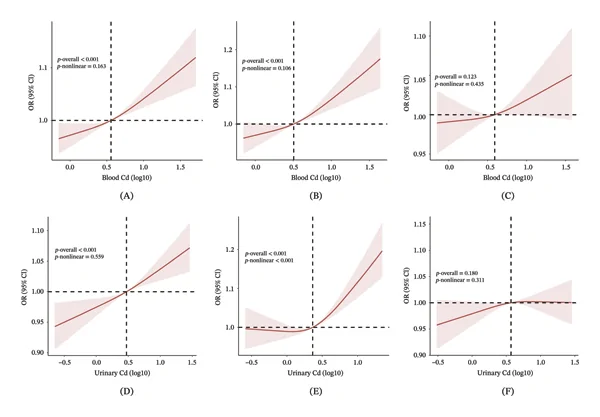
Dose–response relationship between Cd levels and CVD risk. (A): RCS analysis of blood Cd levels and risk of CVD in the general population, (B) RCS analysis of blood Cd levels and risk of CVD in males, (C) RCS analysis of blood Cd levels and risk of CVD in females, (D) RCS analysis of urinary Cd levels and risk of CVD in the general population, (E) RCS analysis of urinary Cd levels and risk of CVD in males, and (F) RCS analysis of urinary Cd levels and risk of CVD in females.

### 3.4. Exploratory Mediating Effect of NLR on the Association Between Cd and CVD Risk

Previous univariate logistic regression analysis identified NLR, SII, and NPAR as significant influencing factors for CVD. To further explore this relationship, we assessed the dose–response relationship between NLR levels and CVD risk (Table 5). Multivariate logistic regression analysis revealed that elevated NLR levels were significantly associated with higher CVD prevalence (Table 5). Compared to the Q1 group, higher NLR levels in the Q4 group were associated with higher CVD prevalence in both the general population (OR = 2.51, 95% CI: 1.60–3.94, p < 0.001) and in males (OR = 2.55, 95% CI: 1.64–3.96, p < 0.001), with significant upward trends (p < 0.001 for the general population and p < 0.001 for males). In females, the Q4 group also showed higher CVD prevalence (OR = 3.68, 95% CI: 1.68–8.06, p = 0.001); however, the dose–response trend across quartiles was less pronounced (p = 0.005 for females). However, after multivariable adjustment, SII and NPAR were not found to be significantly associated with CVD risk (see Supporting Information available here). Furthermore, RCS analysis revealed a linear dose–response relationship between NLR levels and CVD risk in the general population (*p*_overall_ < 0.001, *p*_nonlinear_ = 0.527), in males (*p*_overall_ = 0.032, *p*_nonlinear_ = 0.918), and in females (*p*_overall_ < 0.001, *p*_nonlinear_ = 0.198) (see Supporting Information available here).

**TABLE 5:** OR (95% CI) of CVD under different quantiles of NLR.

Participants	NLR	Model 1	*p* value	Model 2	*p* value	Model 3	*p* value
Total	Q1 (< 1.47)	1 (reference)		1 (reference)		1 (reference)	
	Q2 (1.47–1.95)	1.17 (0.69–1.99)	0.568	1.28 (0.74–2.21)	0.370	1.32 (0.76–2.30)	0.320
	Q3 (1.95–2.66)	1.78 (1.14–2.77)	0.012	1.65 (1.03–2.63)	0.038	1.64 (1.00–2.70)	0.051
	Q4 (> 2.66)	3.16 (2.06–4.83)	< 0.001	2.62 (1.68–4.10)	< 0.001	2.51 (1.60–3.94)	< 0.001
	*p*_trend_	< 0.001		< 0.001		< 0.001	

Male	Q1 (< 1.47)	1 (reference)		1 (reference)		1 (reference)	
	Q2 (1.47–1.97)	1.47 (0.68–3.16)	0.321	1.38 (0.63–3.03)	0.415	1.31 (0.75–2.30)	0.334
	Q3 (1.97–2.69)	2.28 (1.18–4.40)	0.014	1.81 (0.91–3.58)	0.091	1.66 (1.01–2.73)	0.045
	Q4 (> 2.69)	3.75 (2.15–6.53)	< 0.001	2.34 (1.25–4.38)	0.008	2.55 (1.64–3.96)	< 0.001
	*p*_trend_	< 0.001		0.032		< 0.001	

Female	Q1 (< 1.47)	1 (reference)		1 (reference)		1 (reference)	
	Q2 (1.47–1.94)	0.92 (0.48–1.78)	0.803	1.11 (0.59–2.12)	0.739	1.37 (0.70–2.68)	0.355
	Q3 (1.94–2.64)	1.24 (0.63–2.43)	0.537	1.35 (0.68–2.68)	0.393	1.82 (0.85–3.87)	0.121
	Q4 (> 2.64)	2.60 (1.32–5.10)	0.006	2.86 (1.48–5.53)	0.002	3.68 (1.68–8.06)	0.001
	*p*_trend_	0.001		0.003		0.005	

*Note:* Model 1: unadjusted; Model 2: adjusted for age; Model 3: additionally adjusted for education level, race, smoking status, drinking status, hypertension, diabetes mellitus, family history of CHD, TC, TG, LDL‐C, HDL‐C, and UA.

We further examined the mediating effects of NLR in the relationship between Cd levels and CVD risk (Figure 2). The exploratory analysis revealed that NLR may partly explain the observed cross‐sectional association between Cd exposure and CVD risk. Specifically, the NLR‐mediated effect accounted for 7.2% (95% CI: 2.2%–12.2%) of the association between blood Cd and CVD risk in the general population, and 9.4% (95% CI: 3.5%–15.2%) in males. Similar results were observed for urinary Cd, with NLR‐mediated effects accounting for 6.4% (95% CI: 2.8%–10.0%) in the general population and 5.0% (95% CI: 1.9%–8.1%) in males (Table 6). In this framework, the total effect equals the sum of the direct and indirect effects; thus, a detectable total effect is a prerequisite for interpreting any decomposed pathways. In females, blood Cd did not exhibit a significant total effect on CVD risk (Table 2; *p* = 0.50 > 5). Although urinary Cd showed a borderline significant association (Table 3; *p* = 0.046), the total effect from the survey‐design‐compatible probit model was 0.428 (95% CI: 0.169‐0.687). Given the lack of a robust total effect and the cross‐sectional design, mediation estimates for females are provided for transparency but are not interpreted in the main text.

**FIGURE 2:**
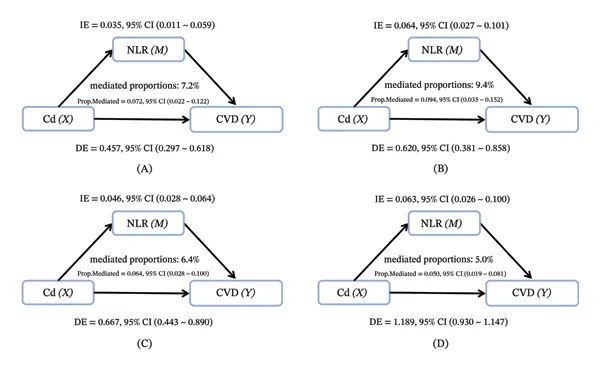
Exploratory mediating effect of NLR on the Cd‐associated cross‐sectional association with CVD risk. (A) Exploratory mediating effect of NLR on the blood Cd–associated cross‐sectional association with CVD risk in the general population, (B) exploratory mediating effect of NLR on the blood Cd–associated cross‐sectional association with CVD risk in males, (C) exploratory mediating effect of NLR on the urinary Cd–associated cross‐sectional association with CVD risk in the general population, and (D) exploratory mediating effect of NLR on the urinary Cd–associated cross‐sectional association with CVD risk in males.

**TABLE 6:** Exploratory mediation analysis of the cross‐sectional associations between cadmium exposure and CVD.

Exposure	Group	Total effect (95% CI)	Direct effect (95% CI)	Indirect effect (95% CI)	Relative effect (%)
Blood Cd	Overall	0.493 (0.328, 0.657)	0.457 (0.297, 0.618)	0.035 (0.011, 0.059)	7.2 (2.2%, 12.2)
	Male	0.684 (0.440, 0.927)	0.620 (0.381, 0.858)	0.064 (0.027, 0.101)	9.4 (3.5%, 15.2)
	Female	0.261 (−0.019, 0.502)	0.260 (0.026, 0.495)	0.000 (−0.042, 0.042)	0.1 (−73.5%,73.7)

Urinary Cd	Overall	0.713 (0.495, 0.931)	0.667 (0.443, 0.890)	0.046 (0.028, 0.064)	6.4 (2.8%, 10.0)
	Male	1.251 (0.993, 1.510)	1.189 (0.930, 1.447)	0.063 (0.026, 0.100)	5.0 (1.9%, 8.1)
	Female	0.428 (0.169, 0.687)	0.404 (0.128, 0.679)	0.025 (−0.009, 0.059)	5.8 (−3.9%, 15.5)

*Note:* Data are *β* coefficients and 95% confidence intervals (CIs). The indirect effect represents the exploratory mediating effect, and the relative effect indicates the proportion mediated. Estimates for females are provided for transparency but should be interpreted with caution. For females, the total effect of blood Cd was not statistically significant (95% CI includes 0), and the total effect of urinary Cd, while nominally significant in the probit model, was not robust in the logistic regression (Table 3; *p* = 0.046 with wide CI). Therefore, mediation estimates for females are provided for transparency but should be interpreted with caution and are not discussed in the main text. All mediation findings are exploratory due to the cross‐sectional design and should not be interpreted as causal.

## 4. Discussion

CVD is a leading cause of death, with its high morbidity and mortality posing a serious threat to human life and health. Although existing clinical treatment strategies have made some progress, these approaches primarily focus on alleviating symptoms, preventing complications, and reducing acute mortality, without fundamentally altering the pathological processes of CVD [20]. As a result, preventing the occurrence of CVD remains crucial. Common cardiovascular risk factors include hypertension, hyperlipidemia, DM, and smoking [21–24]. Despite the identification and management of many of these risk factors, the effectiveness of CVD prevention remains suboptimal. Therefore, there is an urgent need to identify new potential risk factors to enhance the prevention and control of CVD. Our cross‐sectional study identified significant cross‐sectional associations of Cd and NLR with CVD, particularly in males, both of which were cross‐sectionally associated with higher CVD prevalence. However, the cross‐sectional design precludes causal inference. This study found that both blood Cd and urinary Cd were significantly cross‐sectionally associated with increased odds of CVD.

Compared with low levels of blood Cd and urinary Cd, high levels of blood Cd (OR = 2.60, 95% CI: 1.55–4.37) and urinary Cd (OR = 3.22, 95% CI: 1.76–5.98) were significantly associated with higher CVD prevalence. Furthermore, a dose–response relationship between Cd exposure and CVD risk was explored using RCS analysis. The results indicated a linear dose–response relationship between both blood Cd and urinary Cd levels and CVD risk. Specifically, when blood Cd levels reached 1 μg/L, the odds of CVD were significantly higher (OR = 2.58, 95% CI: 1.78–3.74), which is consistent with our findings [6]. Additionally, their results showed that urinary Cd levels were cross‐sectionally associated with higher CVD prevalence at early stages, but when urinary Cd levels reached 0.5 μg/g, the risk of CVD peaked, which slightly differs from the linear dose–response relationship observed in our study [6]. In the sex‐specific analysis, we found that only in males, high levels of blood Cd (OR = 3.06, 95% CI: 1.45–6.46) and urinary Cd (OR = 3.02, 95% CI: 1.52–6.02) were significantly cross‐sectionally associated with higher CVD prevalence. A significant positive linear dose–response association was observed between blood Cd and CVD risk (*p*_overall_ < 0.001), which remained linear after testing for nonlinearity (*p*_nonlinear_ = 0.163). Similarly, urinary Cd exhibited a significant positive association with CVD risk (*p*_overall_ < 0.001), but there was no statistically significant evidence of a nonlinear association (*p*_nonlinear_ = 0.559).

Borne et al. [25] reported that blood Cd levels were associated with the risk of death from HF in males but not in females. Additionally, a study from Japan found that elevated urinary Cd levels were associated with higher CVD mortality in both males and females, but the risk in males was significantly higher than in females [26]. These findings suggest that the effects of Cd on cardiovascular health may vary by sex. Males might be exposed to more sources of Cd (such as occupational exposure and smoking), leading to higher body concentrations of Cd, potentially increasing their CVD risk with long‐term exposure [27, 28]. In contrast, females may benefit from the protective effects of estrogen, which could reduce the risk of CVD by providing antioxidant and anti‐inflammatory effects on blood vessels [29, 30].

Neutrophils play a crucial role in the body’s immune response, particularly in the inflammatory process, while lymphocytes are primarily involved in anti‐inflammatory activities. An elevated NLR typically reflects an increased systemic inflammatory response [31]. Chronic low‐grade inflammation is considered a key factor in the development of CVD, including CHD, MI, and atherosclerosis [32–34]. Numerous studies have shown that CVD patients have significantly higher NLR levels compared to healthy individuals, and the increase in NLR is closely associated with poor prognosis in CVD patients [35]. In our study, we found that in males, elevated NLR was significantly cross‐sectionally associated with higher CVD prevalence (OR = 2.55, 95% CI: 1.64–3.96), and a positive linear dose–response relationship was observed between NLR levels and CVD risk (*p*_overall_ = 0.032, *p*_nonlinear_ = 0.918). Elevated NLR was cross‐sectionally associated with higher cardiovascular event prevalence; potential mechanisms include impairing endothelial function, promoting inflammatory responses, and accelerating atherosclerosis [34, 36]. Recent studies have shown that Cd exposure is cross‐sectionally associated with systemic inflammatory responses, which may co‐occur with or precede elevated CVD prevalence [37, 38]. As an inflammatory biomarker, NLR has been shown to be associated with Cd exposure and may play a role in Cd‐associated cardiovascular pathophysiology [39, 40]. In exploratory mediation analysis, NLR showed significant indirect effects in males (blood Cd: proportion mediated = 9.4%, 95% CI: 3.5%–15.2%; urinary Cd: 5.0%, 95% CI: 1.9%–8.1%), suggesting that systemic inflammation, as reflected by elevated NLR, may be one of the pathways through which the cross‐sectional association between Cd and CVD risk is partially manifested. Nevertheless, given the cross‐sectional nature of this study, we cannot establish a definitive causal sequence, and reverse causation (e.g., subclinical CVD influencing NLR or health behaviors affecting Cd levels) cannot be ruled out. These mediation findings should be interpreted as hypothesis‐generating pending confirmation in prospective cohorts.

Despite the important findings in this study, several limitations should be acknowledged. First, as a cross‐sectional study, our research provides only a snapshot of the associations between Cd exposure and CVD, which limits the ability to establish causal relationships. Importantly, CVD was defined by self‐reported physician diagnosis prior to the examination, while Cd and NLR were measured at a single time point during the NHANES visit. Therefore, the temporal sequence of Cd exposure ⟶ NLR elevation ⟶ CVD onset cannot be determined, and the exploratory mediation analysis cannot be interpreted as evidence of a causal pathway. Longitudinal, prospective studies would be needed to provide stronger evidence of causality. Second, although we did not observe a significant association between Cd exposure and CVD risk in females, we did not explore the potential influence of hormonal changes before and after menopause on the relationship between Cd and CVD. Future prospective studies will be necessary to further investigate how Cd exposure impacts CVD risk in females at different stages of menopause. Third, while RCS identified a J‐shaped pattern for urinary Cd in males, our primary analysis used predefined quartiles to align with prior literature and facilitate comparison. Sensitivity analyses using the RCS‐derived inflection point yielded consistent effect estimates, but future studies may benefit from data‐driven exposure categorizations.

## 5. Conclusions

In conclusion, this cross‐sectional study investigated the association between Cd exposure and CVD, identifying significant cross‐sectional associations of both blood Cd and urinary Cd with CVD. Higher levels of blood Cd and urinary Cd were significantly associated with increased CVD prevalence, with a clear dose–response relationship observed. Sex‐specific analyses revealed that the impact of Cd exposure on CVD risk was more pronounced in males. Furthermore, this study highlighted the role of NLR as a potential biomarker linked to CVD risk. These cross‐sectional findings suggest a potential interplay between Cd exposure and inflammation, offering hypothesis‐generating insights into the mechanisms underlying CVD that require prospective validation. This study underscores the potential of using NLR as a biomarker for early prediction and intervention in CVD, particularly in male populations exposed to Cd.

## Author Contributions

Xinyi Li, Xiaowei Niu, and Yongbin Lu designed the study; Xinyi Li and Kaiwen Wang wrote the paper; Ruiqi Yang, Peijing Yan, and Hui Xu developed the methodology; Yongbin Lu, Liangying Hou, and Siyu Zhang analyzed the data; Xiaowei Niu and Yongbin Lu supervised and revised the paper.

## Funding

This work was supported by grants from the National Natural Science Foundation of China (No. 82560085), the Science and Technology Program of Gansu Province (No. 24JRRA293), the Foundation for Scientific Research of the Gansu Provincial Talent Program (No. 2025QNTD27), the Lanzhou Municipal Innovation Project for Young Scientific and Technological Talents (No. 2025‐QN‐123), the Fundamental Research Funds for the Central Universities (No. lzujbky‐2024‐pd01), and the Foundation for Scientific Research of the First Hospital of Lanzhou University (No. ldyyyn2025‐235), the Academic Excellence Program of the First Clinical Medical School of Lanzhou University (20250060033).

## Disclosure

All authors have read and approved the final manuscript for publication.

## Ethics Statement

The NHANES survey protocol was approved by the National Center for Health Statistics (NCHS) Ethics Review Board (ERB), and informed consent was obtained from all participants. The present analysis used deidentified public‐use data and was, therefore, exempt from additional institutional review board review.

## Conflicts of Interest

The authors declare no conflicts of interest.

## Supporting Information

Additional supporting information can be found online in the Supporting Information section.

## Supporting information


**Supporting Information 1** The following Supporting information is available: Supporting 1. Supporting Figure S1: Flowchart of participant selection from NHANES 1999–2020.

**Supporting Information 2** Supporting 2. Supporting Figure S2: Restricted cubic spline analysis of neutrophil‐to‐lymphocyte ratio levels with cardiovascular disease risk in the general population, males, and females.

**Supporting Information 3** Supporting Table S1: Univariate logistic regression analysis of CVD risk.

**Supporting Information 4** Supporting Table S2: OR (95% CI) of CVD under different quantiles of SII.

**Supporting Information 5** Supporting Table S3: OR (95% CI) of CVD under different quantiles of NPAR.

**Supporting Information 6** Supporting Table S4: OR (95% CI) of MI under different quantiles of blood Cd.

**Supporting Information 7** Supporting Table S5: OR (95% CI) of MI under different quantiles of urinary Cd.

**Supporting Information 8** Supporting Table S6: OR (95% CI) of HF under different quantiles of blood Cd.

**Supporting Information 9** Supporting Table S7: OR (95% CI) of HF under different quantiles of urinary Cd.

**Supporting Information 10** Supporting Table S8: OR (95% CI) of SAP under different quantiles of blood Cd.

**Supporting Information 11** Supporting Table S9: OR (95% CI) of SAP under different quantiles of urinary Cd.

**Supporting Information 12** Supporting Table S10: OR (95% CI) of CHD under different quantiles of blood Cd.

**Supporting Information 13** Supporting Table S11: OR (95% CI) of CHD under different quantiles of urinary Cd.

## Data Availability

Publicly available datasets were analyzed in this study. These data can be found at https://www.cdc.gov/nchs/nhanes/?CDC_AAref_Val=https://www.cdc.gov/nchs/nhanes/index.htm.
