# Supplementary material for: Sex‐Specific Cross‐Sectional Associations and Exploratory Mediation Analysis of Cardiovascular Disease Risk by Neutrophil‐to‐Lymphocyte Ratio in Cadmium‐Exposed Populations
Source: Cardiol Res Pract. 2026 Oct 21;2026:3957573. doi: 10.1155/crp/3957573 (PMC13687931; doi:10.1155/crp/3957573)
Supplement: Supplementary file 1 [file CRP-2026-3957573-s001.docx]

**Supplementary Table 1 Univariate logistic regression analysis of CVD risk**

| **Stratified analyses** | **Total** | |  | **Male** | |  | **Female** | |
| --- | --- | --- | --- | --- | --- | --- | --- | --- |
|  | **OR (95% CI)** | ***p-value*** |  | **OR (95% CI)** | ***p-value*** |  | **OR (95% CI)** | ***p-value*** |
| **Age group, years** |  |  |  |  |  |  |  |  |
| <60 | 1 |  |  | 1 |  |  | 1 |  |
| >=60 | 7.02(5.41-9.12) | <0.001 |  | 9.02(6.06-13.40) | <0.001 |  | 5.20(3.50-7.74) | <0.001 |
| **Race/ethnicity** |  |  |  |  |  |  |  |  |
| Mexican American | 1 |  |  | 1 |  |  | 1 |  |
| Non-hispanic white | 2.28(1.59-3.26) | <0.001 |  | 2.90(1.92-4.39) | <0.001 |  | 1.67(0.87-3.21) | 0.125 |
| Non-hispanic black | 1.75(1.14-2.69) | 0.011 |  | 1.59(0.88-2.85) | 0.123 |  | 1.97(1.01-3.85) | 0.048 |
| Other | 1.73(1.02-2.92) | 0.043 |  | 1.50(0.79-2.85) | 0.218 |  | 2.03(0.92-4.47) | 0.079 |
| **Education** |  |  |  |  |  |  |  |  |
| Less than high school degree | 1 |  |  | 1 |  |  | 1 |  |
| High school degree | 0.72(0.48-1.08) | 0.111 |  | 0.70(0.40-1.24) | 0.221 |  | 0.74(0.43-1.27) | 0.274 |
| More than high school degree | 0.46(0.32-0.67) | <0.001 |  | 0.61(0.42-0.90) | 0.012 |  | 0.29(0.15-0.56) | <0.001 |
| **BMI** |  |  |  |  |  |  |  |  |
| <25 | 1 |  |  | 1 |  |  | 1 |  |
| 25-30 | 1.76(1.22-2.52) | 0.003 |  | 1.76(1.22-2.52) | 0.003 |  | 2.10(1.12-3.95) | 0.022 |
| >=30 | 1.88(1.43-2.46) | <0.001 |  | 1.88(1.43-2.46) | <0.001 |  | 1.90(1.19-3.04) | 0.007 |
| **Smoking status** |  |  |  |  |  |  |  |  |
| Never smoker | 1 |  |  | 1 |  |  | 1 |  |
| Ever smoker | 2.59(1.99-3.38) | <0.001 |  | 4.52(3.06-6.67) | <0.001 |  | 1.21(0.72-2.05) | 0.464 |
| Current smoker | 1.35(0.82-1.96) | 0.122 |  | 2.30(1.41-3.76) | 0.001 |  | 0.59(0.34-1.02) | 0.059 |
| **Drinking status** |  |  |  |  |  |  |  |  |
| Never drinker | 1 |  |  | 1 |  |  | 1 |  |
| Ever drinker | 1.72(1.05-2.80) | 0.031 |  | 1.06(0.48-2.35) | 0.879 |  | 2.77(1.59-4.80) | <0.001 |
| Current drinker | 0.85(0.38-1.88) | 0.681 |  | 1.03(0.29-3.62) | 0.965 |  | 0.94(0.34-2.62) | 0.904 |
| **Physical exercise** |  |  |  |  |  |  |  |  |
| Never | 1 |  |  | 1 |  |  | 1 |  |
| Occasionally | 0.71(0.48-1.07) | 0.1 |  | 0.74(0.44-1.26) | 0.265 |  | 0.67(0.35-1.30) | 0.235 |
| Offen | 1.05(0.68-1.63) | 0.827 |  | 0.99(0.55-1.77) | 0.976 |  | 1.20(0.61-2.35) | 0.602 |
| **hypertension** |  |  |  |  |  |  |  |  |
| No | 1 |  |  | 1 |  |  | 1 |  |
| Yes | 4.67(3.38-6.46) | <0.001 |  | 5.20(3.39-7.97) | <0.001 |  | 4.04(2.48-6.59) | <0.001 |
| **Diabetes mellitus** |  |  |  |  |  |  |  |  |
| No | 1 |  |  | 1 |  |  | 1 |  |
| Yes | 3.56(2.61-4.87) | <0.001 |  | 2.97(1.95-4.50) | <0.001 |  | 4.30(2.75-6.72) | <0.001 |
| **Hyperlipemia** |  |  |  |  |  |  |  |  |
| No | 1 |  |  | 1 |  |  | 1 |  |
| Yes | 1.40(1.03-1.91) | 0.031 |  | 1.14(0.76-1.69) | 0.528 |  | 1.62(0.97-2.69) | 0.064 |
| **Hyperuricemia, mg/dL** |  |  |  |  |  |  |  |  |
| <6/7 (male/female) | 1 |  |  | 1 |  |  | 1 |  |
| >=6/7 (male/female) | 1.90(1.37-2.64) | <0.001 |  | 1.36(0.93-1.99) | 0.107 |  | 2.81(1.82-4.32) | <0.001 |
| **Family history of CHD** |  |  |  |  |  |  |  |  |
| No | 1 |  |  | 1 |  |  | 1 |  |
| Yes | 2.86(2.01-4.08) | <0.001 |  | 2.59(1.69-3.96) | <0.001 |  | 3.46(2.06-5.81) | <0.001 |
| **TC, mmol/L** |  |  |  |  |  |  |  |  |
| Normal | 1 |  |  | 1 |  |  | 1 |  |
| High | 0.79(0.45-1.37) | 0.396 |  | 0.52(0.28-0.98) | 0.043 |  | 1.25(0.57-2.72) | 0.571 |
| **TG, mmol/L** |  |  |  |  |  |  |  |  |
| Normal | 1 |  |  | 1 |  |  | 1 |  |
| High | 0.51(0.36-0.72) | <0.001 |  | 0.37(0.24-0.57) | <0.001 |  | 0.85(0.52-1.41) | 0.533 |
| **LDL-C, mmol/L** |  |  |  |  |  |  |  |  |
| Normal | 1 |  |  | 1 |  |  | 1 |  |
| High | 0.76(0.42-1.39) | 0.373 |  | 0.46(0.24-0.87) | 0.018 |  | 1.34(0.54-3.30) | 0.527 |
| **HDL-C, mmol/L** |  |  |  |  |  |  |  |  |
| Normal | 1 |  |  | 1 |  |  | 1 |  |
| High | 0.56(0.38-0.83) | 0.004 |  | 0.64(0.40-1.02) | 0.062 |  | 0.66(0.33-1.31) | 0.228 |
| **CRP, mg/dL** |  |  |  |  |  |  |  |  |
| Normal | 1 |  |  | 1 |  |  | 1 |  |
| High | 1.74(0.97-3.14) | 0.064 |  | 2.48(0.97-6.36) | 0.058 |  | 1.13(0.51-2.49) | 0.764 |
| **NLR** |  |  |  |  |  |  |  |  |
| Normal | 1 |  |  | 1 |  |  | 1 |  |
| High | 2.20(1.62-2.98) | <0.001 |  | 2.37(1.61-3.48) | <0.001 |  | 1.96(1.27-3.03) | 0.003 |
| **SII** |  |  |  |  |  |  |  |  |
| Normal | 1 |  |  | 1 |  |  | 1 |  |
| High | 1.54(1.15-2.07) | 0.004 |  | 1.71(1.14-2.57) | 0.010 |  | 1.53(1.00-2.36) | 0.049 |
| **PLR** |  |  |  |  |  |  |  |  |
| Normal | 1 |  |  | 1 |  |  | 1 |  |
| High | 0.91(0.64-1.29) | 0.607 |  | 0.89(0.55-1.46) | 0.646 |  | 1.16(0.75-1.78) | 0.505 |
| **NPAR** |  |  |  |  |  |  |  |  |
| Normal | 1 |  |  | 1 |  |  | 1 |  |
| High | 0.43(0.32-0.57) | <0.001 |  | 0.35(0.24-0.52) | <0.001 |  | 0.60(0.37-0.96) | 0.031 |
| **Blood Cd,ug/L** |  |  |  |  |  |  |  |  |
| Normal | 1 |  |  | 1 |  |  | 1 |  |
| High | 1.59(1.05-2.41) | 0.027 |  | 1.85(1.10-3.09) | 0.020 |  | 1.53(0.76-3.08) | 0.226 |
| **Urinary Cd,ug/g** |  |  |  |  |  |  |  |  |
| Normal | 1 |  |  | 1 |  |  | 1 |  |
| High | 2.81(1.93-4.12) | <0.001 |  | 4.63(2.95-7.25) | <0.001 |  | 1.98(1.15-3.40) | 0.014 |
