# Supplementary material for: Sex‐Specific Cross‐Sectional Associations and Exploratory Mediation Analysis of Cardiovascular Disease Risk by Neutrophil‐to‐Lymphocyte Ratio in Cadmium‐Exposed Populations
Source: Cardiol Res Pract. 2026 Oct 21;2026:3957573. doi: 10.1155/crp/3957573 (PMC13687931; doi:10.1155/crp/3957573)
Supplement: Supplementary file 2 [file CRP-2026-3957573-s002.docx]

**Supplementary Table 2 OR (95% CI) of CVD under different quantiles of SII**

| **Participants** | **SII** | **Model 1** | ***p-value*** | **Model 2** | ***p-value*** | **Model 3** | ***p-value*** |
| --- | --- | --- | --- | --- | --- | --- | --- |
| **Total** | Q1(<342.11) | 1(Reference) |  | 1(Reference) |  | 1(Reference) |  |
|  | Q2(342.11-477.43) | 0.90(0.61-1.31) | 0.568 | 0.90(0.60-1.37) | 0.629 | 0.88(0.56-1.38) | 0.570 |
|  | Q3(477.43-684.99) | 1.32(0.91-1.92) | 0.140 | 1.31(0.89-1.95) | 0.172 | 1.21(0.81-1.79) | 0.349 |
|  | Q4(>684.99) | 1.61(1.10-2.37) | 0.015 | 1.67(1.11-2.50) | 0.013 | 1.41(0.89-2.23) | 0.137 |
|  | *P_trend_* | 0.042 |  | 0.042 |  | 0.200 |  |
| **Male** | Q1(<325.44) | 1(Reference) |  | 1(Reference) |  | 1(Reference) |  |
|  | Q2(325.44-448.68) | 0.85(0.49-1.46) | 0.546 | 0.76(0.43-1.35) | 0.352 | 0.87(0.55-1.37) | 0.532 |
|  | Q3(448.68-635.09) | 1.41(0.81-2.44) | 0.222 | 1.08(0.61-1.91) | 0.792 | 1.20(0.81-1.78) | 0.364 |
|  | Q4(>635.09) | 1.93(1.11-3.35) | 0.019 | 1.46(0.80-2.69) | 0.218 | 1.36(0.86-2.17) | 0.187 |
|  | *P_trend_* | 0.030 |  | 0.300 |  | 0.300 |  |
| **Female** | Q1(<361.13) | 1(Reference) |  | 1(Reference) |  | 1(Reference) |  |
|  | Q2(361.13-504.58) | 1.09(0.57-2.07) | 0.796 | 1.19(0.61-2.35) | 0.606 | 1.26(0.62-2.56) | 0.526 |
|  | Q3(504.58-736.47) | 1.41(0.75-2.66) | 0.287 | 1.74(0.93-3.24) | 0.083 | 2.05(1.02-4.12) | 0.045 |
|  | Q4(>736.47) | 1.67(0.94-2.98) | 0.080 | 2.01(1.09-3.70) | 0.026 | 2.08(1.03-4.22) | 0.042 |
|  | *P_trend_* | 0.300 |  | 0.060 |  | 0.064 |  |

Model 1: Unadjusted; Model 2: Adjust for age; Model 3: Additionally adjust for education level, race, smoking status, drinking status, hypertension, diabetes mellitus, Family history of CHD, TC, TG, LDL-C, HDL-C, UA.
