# Supplementary material for: Sex‐Specific Cross‐Sectional Associations and Exploratory Mediation Analysis of Cardiovascular Disease Risk by Neutrophil‐to‐Lymphocyte Ratio in Cadmium‐Exposed Populations
Source: Cardiol Res Pract. 2026 Oct 21;2026:3957573. doi: 10.1155/crp/3957573 (PMC13687931; doi:10.1155/crp/3957573)
Supplement: Supplementary file 3 [file CRP-2026-3957573-s003.docx]

**Supplementary Table 3 OR (95% CI) of CVD under different quantiles of NPAR**

| **Participants** | **NPAR** | **Model 1** | ***p-value*** | **Model 2** | ***p-value*** | **Model 3** | ***p-value*** |
| --- | --- | --- | --- | --- | --- | --- | --- |
| **Total** | Q1(<3.35) | 1(Reference) |  | 1(Reference) |  | 1(Reference) |  |
|  | Q2(3.35-6.94) | 0.50(0.34-0.73) | <0.001 | 0.56(0.37-0.84) | 0.005 | 0.68(0.43-1.07) | 0.092 |
|  | Q3(6.94-12.73) | 0.50(0.34-0.73) | <0.001 | 0.65(0.43-0.99) | 0.044 | 0.95(0.60-1.50) | 0.811 |
|  | Q4(>12.73) | 0.33(0.21-0.52) | <0.001 | 0.43(0.26-0.69) | <0.001 | 0.63(0.39-1.01) | 0.056 |
|  | *P_trend_* | <0.001 |  | 0.001 |  | 0.200 |  |
| **Male** | Q1(<3.20) | 1(Reference) |  | 1(Reference) |  | 1(Reference) |  |
|  | Q2(3.20-6.54) | 0.36(0.21-0.63) | <0.001 | 0.43(0.24-0.78) | 0.005 | 0.69(0.44-1.08) | 0.101 |
|  | Q3(6.54-11.98) | 0.45(0.28-0.71) | <0.001 | 0.59(0.35-1.00) | 0.051 | 0.95(0.60-1.50) | 0.817 |
|  | Q4(>11.98) | 0.23(0.13-0.38) | <0.001 | 0.30(0.16-0.53) | <0.001 | 0.62(0.38-1.00) | 0.051 |
|  | *P_trend_* | <0.001 |  | <0.001 |  | 0.200 |  |
| **Female** | Q1(<3.53) | 1(Reference) |  | 1(Reference) |  | 1(Reference) |  |
|  | Q2(3.53-7.32) | 0.82(0.46-1.46) | 0.493 | 0.86(0.47-1.58) | 0.623 | 1.22(0.60-2.48) | 0.578 |
|  | Q3(7.32-13.60) | 0.63(0.31-1.26) | 0.187 | 0.78(0.39-1.59) | 0.5 | 1.24(0.55-2.78) | 0.604 |
|  | Q4(>13.60) | 0.57(0.30-1.09) | 0.088 | 0.67(0.34-1.31) | 0.24 | 1.10(0.54-2.25) | 0.791 |
|  | *P_trend_* | 0.300 |  | 0.700 |  | 0.900 |  |

Model 1: Unadjusted; Model 2: Adjust for age; Model 3: Additionally adjust for education level, race, smoking status, drinking status, hypertension, diabetes mellitus, Family history of CHD, TC, TG, LDL-C, HDL-C, UA.
