# Supplementary material for: Sex‐Specific Cross‐Sectional Associations and Exploratory Mediation Analysis of Cardiovascular Disease Risk by Neutrophil‐to‐Lymphocyte Ratio in Cadmium‐Exposed Populations
Source: Cardiol Res Pract. 2026 Oct 21;2026:3957573. doi: 10.1155/crp/3957573 (PMC13687931; doi:10.1155/crp/3957573)
Supplement: Supplementary file 4 [file CRP-2026-3957573-s004.docx]

**Supplementary Table 4 OR (95% CI) of MI under different quantiles of blood Cd**

| **Participants** | **Blood Cd (ug/L)** | **Model 1** | ***p-value*** | **Model 2** | ***p-value*** | **Model 3** | ***p-value*** |
| --- | --- | --- | --- | --- | --- | --- | --- |
| **Total**  **(n=275)** | **Q1(<0.32)**  （n=30） | 1(Reference) |  | 1(Reference) |  | 1(Reference) |  |
|  | **Q2(0.32-0.56)**  （n=51） | 1.81(0.90-3.60) | 0.093 | 1.60(0.79-3.24) | 0.193 | 1.66(0.80-3.43) | 0.169 |
|  | **Q3(0.56-0.78)**  （n=89） | 3.96(2.07-7.57) | <0.001 | 3.46(1.77-6.77) | <0.001 | 3.38(1.74-6.57) | <0.001 |
|  | **Q4(>0.78)**  （n=105） | 5.60(3.10-10.1) | <0.001 | 5.22(2.90-9.42) | <0.001 | 6.25(3.35-11.7) | <0.001 |
|  | *p_trend_* | <0.001 |  | <0.001 |  | <0.001 |  |
| **Male**  **(n=195)** | **Q1(<0.30)**  （n=22） | 1(Reference) |  | 1(Reference) |  | 1(Reference) |  |
|  | **Q2(0.30-0.51)**  （n=35） | 2.09(0.90-4.87) | 0.087 | 1.57(0.66-3.75) | 0.303 | 1.62(0.67-3.93) | 0.280 |
|  | **Q3(0.51-0.78)**  （n=67） | 6.68(3.11-14.4) | <0.001 | 4.72(2.16-10.3) | <0.001 | 4.52(2.03-10.1) | <0.001 |
|  | **Q4(>0.78)**  （n=71） | 7.08(3.26-15.4) | <0.001 | 5.78(2.63-12.7) | <0.001 | 6.69(2.83-15.8) | <0.001 |
|  | *p_trend_* | <0.001 |  | <0.001 |  | <0.001 |  |
| **Female**  **(n=80)** | **Q1(<0.38)**  （n=8） | 1(Reference) |  | 1(Reference) |  | 1(Reference) |  |
|  | **Q2(0.38-0.59)**  （n=16） | 1.68(0.56-5.06) | 0.354 | 1.41(0.45-4.49) | 0.554 | 1.55(0.47-5.15) | 0.470 |
|  | **Q3(0.59-0.78)**  （n=22） | 1.65(0.58-4.67) | 0.346 | 1.10(0.38-3.13) | 0.865 | 1.02(0.36-2.90) | 0.965 |
|  | **Q4(>0.78)**  （n=34） | 4.24(1.55-11.6) | 0.005 | 3.27(1.15-9.29) | 0.027 | 3.36(0.87-13.0) | 0.078 |
|  | *p_trend_* | 0.020 |  | 0.035 |  | 0.200 |  |

Model 1: Unadjusted; Model 2: Adjust for age; Model 3: Additionally adjust for education level, race, smoking status, drinking status, hypertension, diabetes mellitus, Family history of CHD, TC, TG, LDL-C, HDL-C, UA.
