# Supplementary material for: Sex‐Specific Cross‐Sectional Associations and Exploratory Mediation Analysis of Cardiovascular Disease Risk by Neutrophil‐to‐Lymphocyte Ratio in Cadmium‐Exposed Populations
Source: Cardiol Res Pract. 2026 Oct 21;2026:3957573. doi: 10.1155/crp/3957573 (PMC13687931; doi:10.1155/crp/3957573)
Supplement: Supplementary file 5 [file CRP-2026-3957573-s005.docx]

**Supplementary Table 5 OR (95% CI) of MI under different quantiles of urinary Cd**

| **Participants** | **Urinary Cd (ug/g)** | **Model 1** | ***p-value*** | **Model 2** | ***p-value*** | **Model 3** | ***p-value*** |
| --- | --- | --- | --- | --- | --- | --- | --- |
| **Total**  **(n=275)** | **Q1(<0.22)**  （n=28） | 1(Reference) |  | 1(Reference) |  | 1(Reference) |  |
|  | **Q2(0.22-0.47)**  （n=41） | 1.30(0.57-2.97) | <0.001 | 1.25(0.54-2.89) | 0.605 | 1.22(0.51-2.93) | 0.655 |
|  | **Q3(0.47-0.72)**  （n=81） | 3.32(1.53-7.24) | 0.530 | 3.45(1.50-7.94) | 0.004 | 3.46(1.51-7.90) | 0.004 |
|  | **Q4(>0.72)**  （n=125） | 6.40(3.31-12.4) | 0.003 | 6.26(3.02-13.0) | <0.001 | 5.12(2.43-10.8) | <0.001 |
|  | *p_trend_* | <0.001 |  | <0.001 |  | <0.001 |  |
| **Male**  **(n=195)** | **Q1(<0.12)**  （n=26） | 1(Reference) |  | 1(Reference) |  | 1(Reference) |  |
|  | **Q2(0.12-0.36)**  （n=30） | 1.58(0.61-4.07) | 0343 | 1.18(0.46-3.01) | 0.724 | 1.13(0.42-3.03) | 0.813 |
|  | **Q3(0.36-0.62)**  （n=55） | 5.46(2.31-12.9) | <0.001 | 3.67(1.48-9.12) | 0.005 | 3.87(1.61-9.32) | 0.002 |
|  | **Q4(>0.62)**  （n=84） | 12.3(5.87-25.7) | <0.001 | 6.69(3.03-14.7) | <0.001 | 5.05(2.20-11.6) | <0.001 |
|  | *p_trend_* | <0.001 |  | <0.001 |  | <0.001 |  |
| **Female**  **(n=80)** | **Q1(<0.33)**  （n=2） | 1(Reference) |  | 1(Reference) |  | 1(Reference) |  |
|  | **Q2(0.33-0.57)**  （n=11） | 1.67(0.29-9.80) | 0.567 | 1.30(0.22-7.72) | 0.775 | 1.07(0.20-5.78) | 0.940 |
|  | Q**3(0.57-0.80**)  （n=26） | 3.41(0.58-19.9) | 0.171 | 2.08(0.34-12.6) | 0.442 | 1.39(0.25-7.85) | 0.706 |
|  | **Q4(>0.80)**  （n=41） | 7.47(1.39-40.1) | 0.020 | 3.80(0.59-24.5) | 0.158 | 2.83(0.47-16.9) | 0.251 |
|  | *p_trend_* | <0.001 |  | 0.130 |  | 0.300 |  |

Model 1: Unadjusted; Model 2: Adjust for age; Model 3: Additionally adjust for education level, race, smoking status, drinking status, hypertension, diabetes mellitus, Family history of CHD, TC, TG, LDL-C, HDL-C, UA.
