# Supplementary material for: Sex‐Specific Cross‐Sectional Associations and Exploratory Mediation Analysis of Cardiovascular Disease Risk by Neutrophil‐to‐Lymphocyte Ratio in Cadmium‐Exposed Populations
Source: Cardiol Res Pract. 2026 Oct 21;2026:3957573. doi: 10.1155/crp/3957573 (PMC13687931; doi:10.1155/crp/3957573)
Supplement: Supplementary file 6 [file CRP-2026-3957573-s006.docx]

**Supplementary Table 6 OR (95% CI) of HF under different quantiles of blood Cd**

| **Participants** | **Blood Cd (ug/L)** | **Model 1** | ***p-value*** | **Model 2** | ***p-value*** | **Model 3** | ***p-value*** |
| --- | --- | --- | --- | --- | --- | --- | --- |
| **Total**  **(n=186)** | **Q1(<0.32)**  （n=25） | 1(Reference) |  | 1(Reference) |  | 1(Reference) |  |
|  | **Q2(0.32-0.56)**  （n=33） | 0.93(0.50-1.72) | 0.809 | 0.74(0.39-1.40) | 0.346 | 0.78(0.39-1.55) | 0.467 |
|  | **Q3(0.56-0.78)**  （n=55） | 1.89(0.96-3.73) | 0.065 | 1.31(0.65-2.64) | 0.450 | 1.32(0.69-2.53) | 0.403 |
|  | **Q4(>0.78)**  （n=73） | 4.12(2.12-8.03) | <0.001 | 3.28(1.65-6.54) | <0.001 | 3.61(1.77-7.36) | <0.001 |
|  | *p_trend_* | <0.001 |  | <0.001 |  | <0.001 |  |
| **Male**  **(n=105)** | **Q1(<0.30)**  （n=16） | 1(Reference) |  | 1(Reference) |  | 1(Reference) |  |
|  | **Q2(0.30-0.51)**  （n=14） | 0.64(0.24-1.71) | 0.373 | 0.46(0.17-1.25) | 0.125 | 0.45(0.14-1.46) | 0.184 |
|  | **Q3(0.51-0.78)**  （n=35） | 2.30(0.96-5.51) | 0.061 | 1.48(0.61-3.59) | 0.388 | 1.33(0.54-3.27) | 0.537 |
|  | **Q4(>0.78)**  （n=40） | 4.22(1.74-10.3) | 0.002 | 3.23(1.28-8.14) | 0.013 | 3.11(0.96-10.1) | 0.058 |
|  | *p_trend_* | <0.001 |  | <0.001 |  | 0.005 | 0.184 |
| **Female**  **(n=81)** | **Q1(<0.38)**  （n=9） | 1(Reference) |  | 1(Reference) |  | 1(Reference) |  |
|  | **Q2(0.38-0.59)**  （n=19） | 1.46(0.51-4.17) | 0.481 | 1.20(0.41-3.56) | 0.739 | 1.22(0.41-3.59) | 0.721 |
|  | **Q3(0.59-0.78)**  （n=20） | 1.78(0.66-4.82) | 0.256 | 1.14(0.39-3.33) | 0.809 | 1.11(0.40-3.07) | 0.835 |
|  | **Q4(>0.78)**  （n=33） | 4.47(1.74-11.5) | 0.002 | 3.38(1.26-9.07) | 0.016 | 3.80(1.14-12.7) | 0.031 |
|  | *p_trend_* | 0.001 |  | 0.004 |  | 0.038 |  |

Model 1: Unadjusted; Model 2: Adjust for age; Model 3: Additionally adjust for education level, race, smoking status, drinking status, hypertension, diabetes mellitus, Family history of CHD, TC, TG, LDL-C, HDL-C, UA.
