# Supplementary material for: Sex‐Specific Cross‐Sectional Associations and Exploratory Mediation Analysis of Cardiovascular Disease Risk by Neutrophil‐to‐Lymphocyte Ratio in Cadmium‐Exposed Populations
Source: Cardiol Res Pract. 2026 Oct 21;2026:3957573. doi: 10.1155/crp/3957573 (PMC13687931; doi:10.1155/crp/3957573)
Supplement: Supplementary file 7 [file CRP-2026-3957573-s007.docx]

**Supplementary Table 7 OR (95% CI) of HF under different quantiles of urinary Cd**

| **Participants** | **Urinary Cd (ug/g)** | **Model 1** | ***p-value*** | **Model 2** | ***p-value*** | **Model 3** | ***p-value*** |
| --- | --- | --- | --- | --- | --- | --- | --- |
| **Total**  **(n=186)** | **Q1(<0.22)**  （n=17） | 1(Reference) |  | 1(Reference) |  | 1(Reference) |  |
|  | **Q2(0.22-0.47)**  （n=32） | 0.84(0.32-2.18) | 0.712 | 0.67(0.25-1.79) | 0.418 | 0.64(0.23-1.75) | 0.377 |
|  | **Q3(0.47-0.72)**  （n=59） | 3.27(1.39-7.70) | 0.007 | 2.38(0.90-6.31) | 0.080 | 2.15(0.84-5.53) | 0.110 |
|  | **Q4(>0.72)**  （n=78） | 4.15(1.77-9.72) | <0.001 | 2.49(0.92-6.68) | 0.071 | 1.86(0.74-4.66) | 0.184 |
|  | *p_trend_* | <0.001 |  | 0.003 |  | 0.005 |  |
| **Male**  **(n=105)** | **Q1(<0.12)**  （n=13） | 1(Reference) |  | 1(Reference) |  | 1(Reference) |  |
|  | **Q2(0.12-0.36)**  （n=19） | 0.80(0.24-2.67) | 0.712 | 0.57(0.17-1.91) | 0.355 | 0.52(0.16-1.71) | 0.276 |
|  | **Q3(0.36-0.62)**  （n=28） | 4.20(1.58-11.2) | 0.004 | 2.61(0.84-8.09) | 0.096 | 2.43(0.96-6.19) | 0.061 |
|  | **Q4(>0.62)**  （n=45） | 7.37(2.81-19.3) | 0.001 | 3.53(1.26-9.90) | 0.017 | 2.34(1.02-5.39) | 0.046 |
|  | *p_trend_* | <0.001 |  | 0.003 |  | 0.022 |  |
| **Female**  **(n=81)** | **Q1(<0.33)**  （n=4） | 1(Reference) |  | 1(Reference) |  | 1(Reference) |  |
|  | **Q2(0.33-0.57)**  （n=13） | 0.95(0.25-3.58) | 0.944 | 0.68(0.18-2.57) | 0.562 | 0.72(0.16-3.15) | 0.660 |
|  | **Q3(0.57-0.80)**  （n=31） | 2.91(0.87-9.69) | 0.082 | 1.54(0.43-5.49) | 0.506 | 1.37(0.36-5.17) | 0.639 |
|  | **Q4(>0.80)**  （n=33） | 2.81(0.86-9.20) | 0.086 | 1.18(0.29-4.80) | 0.812 | 1.10(0.23-5.31) | 0.907 |
|  | *p_trend_* | 0.014 |  | 0.300 |  | 0.500 |  |

Model 1: Unadjusted; Model 2: Adjust for age; Model 3: Additionally adjust for education level, race, smoking status, drinking status, hypertension, diabetes mellitus, Family history of CHD, TC, TG, LDL-C, HDL-C, UA.
