# Supplementary material for: Sex‐Specific Cross‐Sectional Associations and Exploratory Mediation Analysis of Cardiovascular Disease Risk by Neutrophil‐to‐Lymphocyte Ratio in Cadmium‐Exposed Populations
Source: Cardiol Res Pract. 2026 Oct 21;2026:3957573. doi: 10.1155/crp/3957573 (PMC13687931; doi:10.1155/crp/3957573)
Supplement: Supplementary file 8 [file CRP-2026-3957573-s008.docx]

**Supplementary Table 8 OR (95% CI) of SAP under different quantiles of blood Cd**

| **Participants** | **Blood Cd (ug/L)** | **Model 1** | ***p-value*** | **Model 2** | ***p-value*** | **Model 3** | ***p-value*** |
| --- | --- | --- | --- | --- | --- | --- | --- |
| **Total**  **(n=165)** | **Q1(<0.32)**  （n=27） | 1(Reference) |  | 1(Reference) |  | 1(Reference) |  |
|  | **Q2(0.32-0.56)**  （n=33） | 1.09(0.51-2.37) | 0.818 | 0.96(0.44-2.07) | 0.914 | 0.96(0.47-1.96) | 0.900 |
|  | **Q3(0.56-0.78)**  （n=60） | 2.85(1.34-6.07) | 0.007 | 2.32(1.05-5.14) | 0.039 | 2.26(0.97-5.26) | 0.059 |
|  | **Q4(>0.78)**  （n=45） | 2.50(1.25-5.02) | 0.010 | 2.16(1.05-4.46) | 0.037 | 2.38(1.06-5.36) | 0.036 |
|  | *p_trend_* | 0.002 |  | 0.014 |  | 0.039 |  |
| **Male**  **(n=97)** | **Q1(<0.30)**  （n=16） | 1(Reference) |  | 1(Reference) |  | 1(Reference) |  |
|  | **Q2(0.30-0.51)**  （n=16） | 1.52(0.53-4.31) | 0.431 | 1.17(0.41-3.35) | 0.766 | 1.00(0.40-2.50) | 0.995 |
|  | **Q3(0.51-0.78)**  （n=38） | 4.48(1.83-11.0) | 0.001 | 3.20(1.29-7.90) | 0.012 | 2.62(1.02-6.73) | 0.045 |
|  | **Q4(>0.78)**  （n=27） | 2.96(1.06-8.21) | 0.038 | 2.35(0.80-6.90) | 0.120 | 1.69(0.61-4.69) | 0.310 |
|  | *p_trend_* | 0.004 |  | 0.035 |  | 0.200 |  |
| **Female**  **(n=68)** | **Q1(<0.38)**  （n=11） | 1(Reference) |  | 1(Reference) |  | 1(Reference) |  |
|  | **Q2(0.38-0.59)**  （n=17） | 0.72(0.28-1.85) | 0.494 | 0.65(0.25-1.69) | 0.375 | 0.73(0.29-1.87) | 0.513 |
|  | **Q3(0.59-0.78)**  （n=22） | 1.76(0.58-5.34) | 0.315 | 1.38(0.42-4.49) | 0.595 | 1.47(0.41-5.30) | 0.552 |
|  | **Q4(>0.78)**  （n=18） | 2.09(0.72-6.07) | 0.175 | 1.77(0.59-5.29) | 0.301 | 1.98(0.45-8.81) | 0.366 |
|  | *p_trend_* | 0.025 |  | 0.060 |  | 0.400 |  |

Model 1: Unadjusted; Model 2: Adjust for age; Model 3: Additionally adjust for education level, race, smoking status, drinking status, hypertension, diabetes mellitus, Family history of CHD, TC, TG, LDL-C, HDL-C, UA.
