# Supplementary material for: Sex‐Specific Cross‐Sectional Associations and Exploratory Mediation Analysis of Cardiovascular Disease Risk by Neutrophil‐to‐Lymphocyte Ratio in Cadmium‐Exposed Populations
Source: Cardiol Res Pract. 2026 Oct 21;2026:3957573. doi: 10.1155/crp/3957573 (PMC13687931; doi:10.1155/crp/3957573)
Supplement: Supplementary file 9 [file CRP-2026-3957573-s009.docx]

**Supplementary Table 9 OR (95% CI) of SAP under different quantiles of urinary Cd**

| **Participants** | **Urinary Cd (ug/g)** | **Model 1** | ***p-value*** | **Model 2** | ***p-value*** | **Model 3** | ***p-value*** |
| --- | --- | --- | --- | --- | --- | --- | --- |
| **Total**  **(n=165)** | **Q1(<0.22)**  （n=18） | 1(Reference) |  | 1(Reference) |  | 1(Reference) |  |
|  | **Q2(0.22-0.47)**  （n=27） | 2.11(0.61-7.31) | 0.235 | 1.96(0.58-6.62) | 0.277 | 1.96(0.57-6.75) | 0.281 |
|  | **Q3(0.47-0.72)**  （n=48） | 3.27(1.34-7.99) | 0.010 | 2.93(1.21-7.10) | 0.018 | 3.05(1.28-7.29) | 0.013 |
|  | **Q4(>0.72)**  （n=72） | 5.66(2.54-12.6) | <0.001 | 4.52(1.93-10.6) | <0.001 | 4.22(1.75-10.2) | 0.002 |
|  | *p_trend_* | <0.001 |  | <0.001 |  | 0.003 |  |
| **Male**  **(n=97)** | **Q1(<0.12)**  （n=16） | 1(Reference) |  | 1(Reference) |  | 1(Reference) |  |
|  | **Q2(0.12-0.36)**  （n=15） | 1.39(0.44-4.41) | 0.576 | 1.07(0.35-3.22) | 0.910 | 0.98(0.30-3.17) | 0.967 |
|  | **Q3(0.36-0.62)**  （n=28） | 3.70(1.40-9.76) | 0.009 | 2.52(0.89-7.15) | 0.081 | 2.83(1.05-7.66) | 0.041 |
|  | **Q4(>0.62)**  （n=38） | 7.85(3.27-18.8) | <0.001 | 4.32(1.52-12.3) | 0.006 | 2.98(1.05-8.51) | 0.041 |
|  | *p_trend_* | <0.001 |  | 0.016 |  | 0.072 |  |
| **Female**  **(n=68)** | **Q1(<0.33)**  （n=2） | 1(Reference) |  | 1(Reference) |  | 1(Reference) |  |
|  | **Q2(0.33-0.57)**  （n=12） | 14.9(2.33-95.5) | 0.005 | 12.9(1.97-84.9) | 0.008 | 12.9(1.98-84.5) | 0.008 |
|  | Q**3(0.57-0.80**)  （n=20） | 13.7(2.82-66.6) | 0.001 | 10.1(2.06-50.1) | 0.005 | 7.62(1.38-42.1) | 0.020 |
|  | **Q4(>0.80)**  （n=34） | 21.1(4.73-94.3) | <0.001 | 13.8(2.91-65.3) | 0.001 | 14.0(2.66-74.0) | 0.002 |
|  | *p_trend_* | 0.014 |  | 0.011 |  | 0.016 |  |

Model 1: Unadjusted; Model 2: Adjust for age; Model 3: Additionally adjust for education level, race, smoking status, drinking status, hypertension, diabetes mellitus, Family history of CHD, TC, TG, LDL-C, HDL-C, UA.
