# Supplementary material for: Sex‐Specific Cross‐Sectional Associations and Exploratory Mediation Analysis of Cardiovascular Disease Risk by Neutrophil‐to‐Lymphocyte Ratio in Cadmium‐Exposed Populations
Source: Cardiol Res Pract. 2026 Oct 21;2026:3957573. doi: 10.1155/crp/3957573 (PMC13687931; doi:10.1155/crp/3957573)
Supplement: Supplementary file 10 [file CRP-2026-3957573-s012.docx]

**Supplementary Table 11 OR (95% CI) of CHD under different quantiles of urinary Cd**

| **Participants** | **Urinary Cd (ug/g)** | **Model 1** | ***p-value*** | **Model 2** | ***p-value*** | **Model 3** | ***p-value*** |
| --- | --- | --- | --- | --- | --- | --- | --- |
| **Total**  **(n=246)** | **Q1(<0.22)**  （n=32） | 1(Reference) |  | 1(Reference) |  | 1(Reference) |  |
|  | **Q2(0.22-0.47)**  （n=44） | 1.08(0.62-1.87) | 0.776 | 0.91(0.52-1.59) | 0.733 | 0.93(0.51-1.71) | 0.824 |
|  | **Q3(0.47-0.72)**  （n=62） | 2.04(0.96-4.32) | 0.063 | 1.63(0.70-3.82) | 0.258 | 1.77(0.80-3.90) | 0.157 |
|  | **Q4(>0.72)**  （n=108） | 3.71(1.99-6.95) | <0.001 | 2.54(1.14-5.66) | 0.022 | 2.43(1.10-5.35) | 0.028 |
|  | *p_trend_* | <0.001 |  | 0.023 |  | 0.061 |  |
| **Male**  **(n=175)** | **Q1(<0.12)**  （n=32） | 1(Reference) |  | 1(Reference) |  | 1(Reference) |  |
|  | **Q2(0.12-0.36)**  （n=33） | 0.98(0.52-1.84) | 0.946 | 0.64(0.33-1.21) | 0.167 | 0.69(0.33-1.42) | 0.306 |
|  | **Q3(0.36-0.62)**  （n=41） | 2.18(0.94-5.07) | 0.069 | 1.21(0.43-3.36) | 0.716 | 1.51(0.61-3.78) | 0.370 |
|  | **Q4(>0.62)**  （n=69） | 5.05(2.68-9.52) | <0.001 | 2.12(0.88-5.07) | 0.091 | 1.86(0.74-4.69) | 0.187 |
|  | *p_trend_* | <0.001 |  | 0.005 |  | 0.140 |  |
| **Female**  **(n=71)** | **Q1(<0.33)**  （n=0） |  |  |  |  |  |  |
|  | **Q2(0.33-0.57)**  （n=11） | 1(Reference) |  | 1(Reference) |  | 1(Reference) |  |
|  | Q**3(0.57-0.80**)  （n=21） | 1.81(0.62-5.28) | 0.273 | 1.47(0.50-4.34) | 0.484 | 1.13(0.40-3.20) | 0.813 |
|  | **Q4(>0.80)**  （n=39） | 2.84(1.02-7.92) | 0.046 | 1.96(0.65-5.89) | 0.230 | 1.70(0.51-5.729) | 0.388 |
|  | *p_trend_* | <0.001 |  | <0.001 |  | <0.001 |  |

Model 1: Unadjusted; Model 2: Adjust for age; Model 3: Additionally adjust for education level, race, smoking status, drinking status, hypertension, diabetes mellitus, Family history of CHD,TC, TG, LDL-C, HDL-C, UA.

Q1 was not observed in the female subgroup due to low exposure levels (n=0); thus, Q2 served as the reference.
