# Supplementary material for: Sex‐Specific Cross‐Sectional Associations and Exploratory Mediation Analysis of Cardiovascular Disease Risk by Neutrophil‐to‐Lymphocyte Ratio in Cadmium‐Exposed Populations
Source: Cardiol Res Pract. 2026 Oct 21;2026:3957573. doi: 10.1155/crp/3957573 (PMC13687931; doi:10.1155/crp/3957573)
Supplement: Supplementary file 11 [file CRP-2026-3957573-s013.docx]

**Supplementary Table 10 OR (95% CI) of CHD under different quantiles of blood Cd**

| **Participants** | **Blood Cd (ug/L)** | **Model 1** | ***p-value*** | **Model 2** | ***p-value*** | **Model 3** | ***p-value*** |
| --- | --- | --- | --- | --- | --- | --- | --- |
| **Total**  **(n=246)** | **Q1(<0.32)**  （n=43） | 1(Reference) |  | 1(Reference) |  | 1(Reference) |  |
|  | **Q2(0.32-0.56)**  （n=42） | 1.18(0.59-2.33) | 0.638 | 0.97(0.48-1.96) | 0.933 | 0.98(0.51-1.91) | 0.963 |
|  | **Q3(0.56-0.78)**  （n=86） | 2.08(1.17-3.70) | 0.013 | 1.57(0.83-2.96) | 0.165 | 1.51(0.80-2.87) | 0.200 |
|  | **Q4(>0.78)**  （n=75） | 2.23(1.29-3.86) | 0.004 | 1.84(1.04-3.26) | 0.038 | 2.21(1.12-4.34) | 0.023 |
|  | *p_trend_* | 0.016 |  | 0.086 |  | 0.087 |  |
| **Male**  **(n=175)** | **Q1(<0.30)**  （n=34） | 1(Reference) |  | 1(Reference) |  | 1(Reference) |  |
|  | **Q2(0.30-0.51)**  （n=29） | 1.13(0.60-2.15) | 0.705 | 0.79(0.42-1.50) | 0.469 | 0.80(0.44-1.47) | 0.476 |
|  | **Q3(0.51-0.78)**  （n=62） | 2.62(1.40-4.91) | 0.003 | 1.68(0.85-3.30) | 0.132 | 1.65(0.77-3.55) | 0.195 |
|  | **Q4(>0.78)**  （n=50） | 2.23(1.06-4.71) | 0.035 | 1.66(0.77-3.59) | 0.197 | 1.87(0.75-4.67) | 0.179 |
|  | *p_trend_* | 0.020 |  | 0.200 |  | 0.300 | 0.184 |
| **Female**  **(n=71)** | **Q1(<0.38)**  （n=9） | 1(Reference) |  | 1(Reference) |  | 1(Reference) |  |
|  | **Q2(0.38-0.59)**  （n=13） | 1.59(0.32-7.91) | 0.569 | 1.37(0.27-6.96) | 0.704 | 1.32(0.29-6.04) | 0.714 |
|  | **Q3(0.59-0.78)**  （n=24） | 2.05(0.55-7.62) | 0.281 | 1.43(0.39-5.31) | 0.587 | 1.18(0.34-4.09) | 0.791 |
|  | **Q4(>0.78)**  （n=25） | 2.81(0.69-11.3) | 0.146 | 2.22(0.56-8.85) | 0.256 | 1.86(0.39-8.78) | 0.432 |
|  | *p_trend_* | 0.500 |  | 0.600 |  | 0.900 |  |

Model 1: Unadjusted; Model 2: Adjust for age; Model 3: Additionally adjust for education level, race, smoking status, drinking status, hypertension, diabetes mellitus, Family history of CHD, TC, TG, LDL-C, HDL-C, UA.
